# Assessing the ‘Why’ in Volunteering for Refugees: Exploring Volunteer Motivations

**DOI:** 10.1007/s11266-023-00574-y

**Published:** 2023-05-25

**Authors:** Maikel Meijeren, Marcel Lubbers, Peer Scheepers

**Affiliations:** 1 Department of Sociology, Radboud University, Thomas van Aquinostraat 4, 6500 HE Nijmegen, The Netherlands; 2 Department of Interdisciplinary Social Science/ERCOMER, Utrecht University, Padualaan 14, 3584 CH Utrecht, The Netherlands

**Keywords:** Volunteers, Refugees, Motivations, Focus groups, Volunteer functions inventory

## Abstract

This article addresses what motivations volunteers have for volunteering for refugees and whether these motivations differ from or complement motivations to volunteer in general, such as included in the widely used measurement instrument, the Volunteer Function Inventory (VFI). We organized eight focus groups with volunteers for refugees (*N* = 44) and interviewed five involved coordinators, all working in one city in the Netherlands. Results show that humanitarian concerns and social justice were highly relevant for people’s motivations, next to volunteering to obtain or improve knowledge and skills. We find support for the earlier suggested extension of the VFI with the social justice motivation. Next, the current study expands existing analysis on volunteer motivations by identifying four areas that require further attention: (1) volunteers for refugees seek a meaningful role in life; (2) are motivated by the pragmatism of this volunteer work; (3) have emotional reasons; and (4) are motivated by media exposure.

## Introduction

Humanitarian crises mobilize parts of civil society (della Porta, 2018; Zamponi & Bosi, 2018), triggering people by the acuteness of the situation, who are willing to volunteer for others who have fled (Fleischmann & Steinhilper, 2017). Volunteers for refugees are considered to fulfill an important bridge function between the host society and refugees. They are among the first to reach out to refugees on behalf of the host society. What distinguishes volunteering for refugees from other volunteering is that time is given to a vulnerable group of newcomers in society, who are not (necessarily) considered to be part of the national ingroup (e.g., Kende et al., 2017). Given their bridge position, it is relevant to study why they want to volunteer for refugees and what motivations lie beneath the willingness to help. Previous research showed that volunteers for refugees have “a broader and more inclusive scope of justice” (Kals & Strubel, 2017, p. 66). Moreover, volunteers for refugees are motivated by moral convictions (Kende et al., 2017), as “refugees are depicted as representing a moral category, whose support is an ethical duty” (Wyszynski et al., 2020, p. 608). However, what still lacks is a comprehensive overview of the different motivations for volunteering for refugees and whether these correspond to motivations previously already identified to be relevant for volunteering in general. Such motivations for volunteering in general are captured by the measurement instrument the Volunteer Function Inventory (VFI) (Clary et al., 1998b). We aim to understand whether volunteers for refugees share the motivations to volunteer that are included in the VFI or whether they or representatives of involved refugee volunteer organizations emphasize additional motivations. By conducting focus groups with volunteers and interviews with their coordinators of refugee volunteer organizations, we provide a more comprehensive overview of the motivations of volunteers for refugees. We study this in the Netherlands, among volunteers for refugees who mainly fled from Afghanistan in the summer of 2021 when the Taliban came to power.^1^ As shown by Van der Veer (2022), these ad hoc social crises situations activate volunteers to reach out to those in need. Therefore, we pose the following research question: What are motivations to volunteer for refugees and do these motivations differ from or complement the general motivations to volunteer as derived from previous theoretical insights?

In doing so, the contribution of this article lies in identifying (un)popular motivations for volunteering for refugees and in connecting theory around motivational instruments like the VFI with relatively new literature on refugee solidarity and politicization to interpret our results and identify missing motivations. It expands the understanding of volunteering in general with exploring and testing to what extent motivations that are considered key, also hold in the context for volunteering for refugees specifically and whether additional motivations apply. Thus, this study explores how applicable a general, leading motivational framework is in a specific area of volunteering. Moreover, our approach helps unraveling specific motivations of volunteering for refugees and may help involved organizations and municipalities to better understand and/or mobilize their volunteers. For instance, by designing more targeted policies to recruit and retain volunteers.

The article is structured as follows: we start with a theoretical inventory to grasp relevant motivations for volunteering for refugees. Then, we outline our methodological approach, together with a clarifying paragraph on the specific Dutch refugee reception situation. Next, we use the results’ section to solely describe results. We provide theoretical reflections on the results in the subsequent discussion section, and end the manuscript with conclusions regarding the research question, main results, implications for future research and limitations of the study.

## Theoretical Insights on Motives to Volunteer

We start with an inventory of previous research on motivations to volunteer, providing us with theoretical insights on these motivations. Then we select a wide range of particular motivations considered plausibly related to motivations to volunteer for refugees. The Volunteer Function Inventory is point of departure. From other volunteering motivation scales, we derive motivations complementary to the VFI we deem relevant for volunteering for refugees. We use these insights to inform our topic list submitted to focus groups with volunteers for refugees.

### Volunteer Function Inventory

One of the most influential motivational instruments to explain volunteering is the VFI (Clary et al., 1998b). The VFI has been tested in many studies (for a review, see Mannino et al., 2011) and identifies a set of motivations that contribute to volunteer work (Wilson, 2012). It assumes that motivations impel actions, and assesses both self-focused and other-focused reasons as to why people volunteer (Mannino et al., 2011). The VFI also acknowledges that different people may volunteer for different reasons.^2^ In total, the VFI is a 30-item instrument to empirically assess six key motivations in volunteering: *values*, *understanding*, *social*, *career*, *protective* and *enhancement*.

Personal *values* are related to a person’s “altruistic and humanitarian concern for others” (Clary et al., 1998b, p. 1517). It is through acts of compassion that volunteers aim to demonstrate their beliefs and humanitarian values (Wuthnow, 1991). Volunteers for refugees in Calais were particularly motivated by such feelings of compassion (Sandri, 2018). Next, *understanding* is concerned with an individual exercising the opportunity to utilize skills on understanding others as well as to develop new skills (Clary et al., 1998b). It thus refers to volunteering as means of personal growth (Musick & Wilson, 2007) and is found to be an important motivator for refugee volunteers (Milan, 2018). *Social* motivations give volunteers a means “to be with one’s friends or to engage in an activity viewed favorably by important others” (Clary et al., 1998b, p. 1518). Dávila and Díaz-Morales (2009) showed that social motives were only of importance among the oldest age volunteers. *Career* motivations relate to career exploration and enhancement (Clary et al., 1998b). Straightforwardly, young people are most likely to volunteer for this reason, especially when they are about to enter the labor market (Musick & Wilson, 2007). Subsequently, *protective* motivations serve volunteers by reducing feelings of guilt associated with their own fortunate circumstances (Clary et al., 1998b). It has to do with enabling people to deal with inner conflicts, uncertainty about the self, emotional needs and the like (Musick & Wilson, 2007). Therefore, humanitarian aid work can be seen as “a moral fulfillment that provides comfort and satisfaction to one’s own self” (Chouliaraki, 2013, p. 4). Finally, *enhancement* motivations “involve positive strivings of the ego” such that the volunteer seeks to develop a positive affect by growing psychologically (Clary et al., 1998b, p. 1518). Malkki (2015, p. 3), for instance, found an “undeniable neediness” among humanitarian aid workers being fulfilled through their volunteering.

### Complementary Motivations

**Fig. 1:**
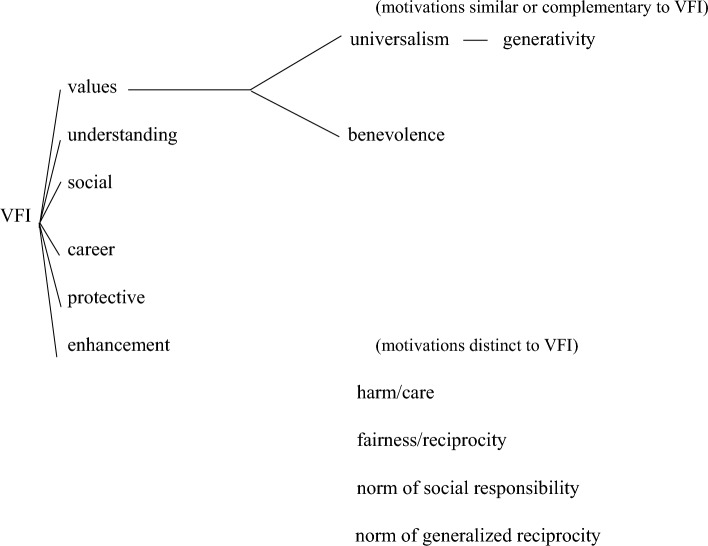
Conceptual map of theoretical insights

There is, however, more research on motivations for volunteering that does not rely on the VFI. That research is discussed here with the aim to discover potential motivations to volunteer for refugees complementary to the VFI. A conceptual map of these additional concepts and how we consider these to relate to the VFI is presented in Fig. 1. Jiranek et al. (2013) also studied additional motives for improving the VFI and added a social justice component. That justice component is also reflected in the *universalism* value of Schwartz (1992; Schwartz et al., 2001).^3^*Universalism* refers to being broadminded and tolerant, expressing societal concern for others in society and showing concern for nature (Schwartz et al., 2001). Two items that represent *universalism* are social justice and equality (Schwartz, 1992). Justice, societal concern, broadmindedness and being tolerant may be relevant to volunteering for refugees. Next, we also select Schwartz’ *benevolence* as these “are also the values most often linked with helping and also volunteering” (Grönlund, 2011, p. 869). *Benevolence* covers actions aimed at improving the welfare of others who need one’s help (Verkasalo et al., 2009). This may apply to volunteering for refugees. Two exemplary items that express *benevolence* are helpfulness and loyalty (Schwartz, 1992). *Universalism* and *benevolence* may be considered part of the VFI *values* function, but are made explicit as separate dimensions by Schwartz.^4^

Next, *generativity* is mentioned in the literature as a possible motivation for people to volunteer and we consider it potentially relevant for volunteering for refugees as well (Musick & Wilson, 2007). *Generativity* points to a concern some people display for the welfare of the next generation and, more general, the welfare of the wider community (Musick & Wilson, 2007). The *Loyola Generativity Scale* (LGS) is developed to measure individual differences in the concern that people have for present and future generations (Morselli & Passini, 2015). Musick and Wilson (2007) found that this generativity predicts volunteering for a wide range of activities and thus might be related to volunteering for refugees as well. LGS relates to VFI *values* and Schwartz’ *universalism* when it comes to concerns about (unknown) others, but is distinct in an explicit concern for the welfare of future generations.

Two other motivations come from Haidt’s (2013) *Moral Foundation Theory* (MFT), where a framework of morality is introduced. MFT states that there are five basic moral foundations, that can be viewed as the building blocks of morality (Prince, 2010). We select *harm/care* and *fairness/reciprocity*, as these foundations may relate most plausibly to volunteering for refugees.^5^*Harm/care* gives rise to specific virtues and vices (Haidt, 2013). Under this foundation, people value kindness and compassion and disapprove cruelty, suffering and aggression. As kindness and compassion are valued by people, it might motivate volunteering for refugees as refugees represent a ‘moral category’ for whom support is an ethical duty that will be rewarded by society (Wyszynski et al., 2020). The need to alleviate the suffering of refugees was the main motivation for many of the volunteers in the study of Monforte and Maestri (2022b). Next, the *fairness/reciprocity* foundation represents the perhaps “most universally recognized virtue–justice” (Prince, 2010, p. 1296). The justice component, as previously added to the VFI by Jiranek et al. (2013), is therefore also reflected in this moral foundation. Hence, *fairness/reciprocity* relates to *universalism* when the importance of justice is considered. As research revealed that volunteers for refugees have a broader and more inclusive scope of justice (Kals & Strubel, 2017; Milan, 2018), this moral foundation is likely to be a relevant motivation in volunteering for refugees.

Further, two norms can serve as motivation in volunteering for refugees.^6^ A norm of *generalized reciprocity* indicates that people provide service to others, or act for the benefit of others, in the generalized expectation that this kindness will be given back at some undefined time by some unknown person, in case of future need (Musick & Wilson, 2007). A norm of *social responsibility* requires people to place community’s wellbeing over personal interests. Volunteers are more likely than non-volunteers to mention that a “good citizen is socially responsible” (Musick & Wilson, 2007, p. 102.). The abstract norm of *generalized reciprocity* deviates from the foregoing, as an explicit appeal is made to the goodness of an unknown person in future. A norm of *social responsibility* approaches the LGS, but is more directed to the own local area instead of wider present and future generations.

## Methodological Strategy

### Data Collection in the Dutch Context

The Central Organization for the Reception of Asylum Seekers (short: COA) is responsible for the reception of refugees and asylum seekers. It is an assignment from the Dutch Ministry of Justice and Safety that is politically responsible. The demand for asylum reception in the Netherlands fluctuates and increased to an estimated 42.000 places by the end of 2022 (COA, 2022a). This aim, however, was set before the war in Ukraine. COA uses five strategies to deal with fluctuations: (1) expanding existing reception centers, reopening reception centers or opening new ones; (2) deploying space capacity at existing centers; (3) deploying temporary capacity, as recreation parks; (4) deploying emergency reception, as refugee camp Heumensoord, where volunteers in this study mainly volunteered; (5) crisis reception in sports halls. COA always (re)opens or closes a reception center only after the local municipality has agreed to it. A municipality is currently not obliged to accommodate newcomers; however, the government works on a law that will oblige municipalities to accommodate newcomers in the future (COA, 2022b). COA works with cooperation partners, such as organizations related to refugees. These organizations have volunteers, who volunteer for refugees under authority of COA. Note that these volunteers themselves are not affiliated to the government. However, as COA is responsible, volunteers must adhere to the guidelines prescribed by COA.

### Background of Refugee Situation and Selection of Volunteers in Focus Groups

In order to host the, approximately, 1000 Afghan refugees who came to the Netherlands, refugee camp Heumensoord was opened by COA in the fall of 2021. This camp is closely located to the Dutch city of Nijmegen, close to the German border. Volunteers were included in this study if they met a number of criteria.^7^ First, we included volunteers who had direct contact with refugees. Second, volunteers had to do their voluntary work in refugee camp Heumensoord, or in Nijmegen. Third, volunteers had to work under the auspice of an involved organization. Selected organizations were the Yalla Foundation Nijmegen and VluchtelingenWerk Nijmegen (Refugee Work Nijmegen). Their selection was based on three criteria: (1) they both had permission to enter the refugee camp; (2) they were willing to cooperate and (3) they could cooperate on short notice (after all, the camp was only temporarily open). Fourth, we only spoke with volunteers who engaged in time consuming regular support initiatives (e.g., Kals & Strubel, 2017), so those who had frequent contact with refugees while volunteering. On the one hand, with permission, volunteer recruitment took place by the authors with calls on the private Yalla Foundation Facebook page. On the other hand, permanent staff of VluchtelingenWerk Nijmegen took care of their recruitment for this study.

We held focus groups with six to eight volunteers per group. Such group sizes are large enough to gain a variety of perspectives and small enough to avoid that focus groups become disorderly or fragmented. Due to strict Covid-19 measures in the Netherlands that were imposed during the data collection, especially during the lockdown in the winter of 2021, three focus groups were conducted online in Zoom. We then chose for a maximum of six participants, to ensure an orderly online environment for optimal data collection. In total, eight focus groups were conducted with a total of 44 participants. Similar to Monforte and Maestri (2022a, 2022b), the majority of the sample was female and older than 50. Females and elderly were overrepresented. Further, all participants were of Dutch nationality. Most volunteers had only recently started volunteering. They were mobilized to volunteer in the temporary refugee camp. Finally, five individual interviews (physical or online) were conducted with coordinators of the involved volunteers.

### Development of Topic Lists for Focus Groups

We developed two separate topic lists: one for the focus groups with volunteers (see appendix A), the other for the individual interviews with coordinators. Note that both lists are identical, with only the difference that coordinators are questioned about their volunteers. In doing so, we apply triangulation as coordinators functioned as ‘third eye’ in the data collection. The focus groups topic list was developed in such a way that participants first were asked about their motivation(s) to volunteer for refugees with an open question. Afterward, participants were referred to existing motivations to volunteer as outlined in the theoretical section, with the use of a small questionnaire. It is with this questionnaire that we aimed to appeal to the intrinsic motivation(s) of participants. Moreover, by using the questionnaire, we were able to ensure that participants could think about motivations that are mentioned in the literature and whether these applied to them. Precisely, participants were questioned which motivations were (un)important in their volunteering, and whether other motivations applied to them that were not included in the questionnaire. With the latter question, we aimed to fully catch all (previously overlooked) motivations to provide a comprehensive overview of motivations for volunteering for refugees. The questionnaire consisted of thirteen items. Every item represented a different motivation. Items one to six referred to, respectively, VFI *career*, *protective*, *understanding*, *enhancement*, *social* and *values*. Item seven measured Schwartz’ *benevolence*, and item eight *universalism*. Item nine referred to LGS. Items ten (*harm/care)* and eleven *(fairness/reciprocity)* represented MFT. Item twelve measured the *generalized reciprocity* norm and item thirteen the norm of *social responsibility*. We had three criteria in the selection of items for the questionnaire: (1) only one item per motivation; (2) sufficient mutual differences between items, to avoid that participants got confused and (3) only include ‘positively’ formulated items. Note that all items were derived from existing and previously validated scales, except for items twelve and thirteen. We chose for one item per motivation only, to not interrupt the dynamic character of a focus group.

### Strategy for Analysis

Texts of focus groups and interviews were transcribed in separate manuscripts. Each manuscript was analyzed from start to finish, to include any motivation that was mentioned more implicitly or appeared during a discussion between participants at any stage of the data collection. Next, by ways of the focus group discussions, people could apply that motive to themselves, even though they did not come up with the motive themselves initially. The latter demonstrates the added value of the focus groups.

The thirteen motives we derived from the literature were coded ‘MO1’ to ‘MO13’. Note that all MO’s correspond with the order of motivations in the questionnaire. We then counted whether a particular motivation was mentioned by any participant, irrespectively of whether it was mentioned multiple times. The counting procedure was as follows: (1) a respondent indicated in the questionnaire certain motivations as important or unimportant and, if applicable; (2) that same respondent mentioned in the focus group a *new* (un)important motivation that was not previously indicated as such in the questionnaire. When participants referred to motivations that were not captured in the questionnaire, and as such were not derived from previous research, we coded them as ‘Missing_MO:_(subject)’. Again, we counted the number of participants that referred to these missing motivations, where also a missing motivation could only count once per respondent. Further, we selected relevant quotes that clarified particular (missing) motivations.

Next, we applied two decision rules: (1) motivations included in the questionnaire were considered popular or unpopular if at least ten participants marked them as such (see supplementary material, appendix B (stored on Figshare.com)); (2) missing motivations would be elaborated upon if they were mentioned at least by five participants (see supplementary material, appendix C (stored on Figshare.com)). Interviews with coordinators served as means to underline the results from volunteers and were used to capture motivations that were not mentioned by volunteers, for instance related to VFI *protective*.

## Results

We find that items of VFI *understanding* and *values* are popular motivations, as well as motivations based on social justice (reflected in Schwartz’ *Universalism* and MFT’s *fairness / reciprocity*). Items of VFI *career* and *social* proved to be unpopular. We identified four missing motivations, being *seeking for a meaningful role*; *pragmatism*; *being driven by media exposure* and *the emotional dimension*. Triangulation identified the importance of VFI *protective*, and also *career*. Below, we systematically elaborate on these results.

### Popular Motivations

The item in the questionnaire *‘Volunteering lets me learn things through direct, hands on experience’* (VFI *understanding*) was the most popular motivation. One volunteer declared: “I'm very interested in other cultures anyway, and it's so enriching to meet those people and see how they deal with their struggles and miseries”.^8^ Also popular was *‘I feel compassion toward people in need’* (VFI *values-*item) and ‘*I volunteer, because I want everyone to be treated justly, even people I don’t know’* (*universalism*). Because this expression of the importance of social justice is also strongly reflected in MFT's *fairness / reciprocity*, we list that moral foundation as popular motivation. A volunteer stated: “Everyone should have equal opportunities, but the people at the bottom of the ladder are the refugees … the moment they put their feet on Dutch ground they have, by definition, an unequal chance and have to go through an institutional jungle. And if I am someone who has to lead them through that jungle, then I want to do that”.^9^ Another volunteer indicated: “I cannot stand it when people have it so lousy. I think that is so terrible”.^10^

### Unpopular Motivations

*‘Volunteering experience will look good on my resume’* (VFI *career*-item) was most unpopular. A volunteer said: “We are here with older people some of whom are already retired, that just does not play a role anymore then, or much less”.^11^ Younger volunteers, however, said: “I am still in the beginning of my career, so that is definitely something that is important to me”.^12^ The other unpopular motive was ‘*people close to me do this type of voluntary work as well’* (VFI *social*-item). Volunteers repeatedly indicated that no one around them did this type of work. However, some volunteers mentioned the role of socialization, which could be considered part of the social function as well, for example: “My grandmother was already taking in Belgian refugees in 1914. And later Hungarian refugees. For me this was normal, I grew up with it. For me it was something very natural. Ordinary, that is what you do”.^13^ Another noteworthy finding was that a few volunteers told that they lost contact with some family members after saying they were doing this volunteer work, or conversely did not tell about this volunteer work for fear of reactions from some of their loved ones. These stories also relate to VFI *social*.

### Missing Motivations

Four missing motivations arose in the analysis. One, volunteers were *seeking for a meaningful role* in life through their volunteering. A volunteer declared: “I started because I just did not want to sit behind the geraniums. And I was looking for something that was useful and that kept me up to date”.^14^ One coordinator described: “helping refugees gives many volunteers also a goal in their lives. They have found a meaning in their life again after retiring from work”.^15^ A second missing motive was the *pragmatism* of volunteers. A volunteer told: “I can exercise my hobby, mean something to those people at the same time and see the immediate results of my actions”.^16^ A third missing motivation was *being driven by media exposure*. Volunteers frequently referred to a situation in a Dutch village in August 2021, where people protested against the refugee arrival. These protests formed the direct motivation for some to start volunteering: “What I also heard a lot was that people wanted to give a counter signal to the protests in Harskamp. Then, of course, you also respond to events shown in the media”.^17^ A last missing motivation was *the emotional dimension*. A volunteer recalled: “Yes, it does affect me. Because if I have a client in front of me, well that could be my father. And that was actually my motive for doing this”.^18^

### Findings from Triangulation

The VFI is often used in (anonymous) questionnaires. It is possible that focus groups are more prone to social desirability bias. To overcome this issue, we applied triangulation by interviews with coordinators. An important aspect that was signaled by coordinators was volunteering to feel less lonely, an item of VFI *protective*, from which we had chosen an item that was formulated positively. One coordinator stated: “I also have some people who did not say that literally, but in which I really saw that they just needed it. People in whom I did see all sorts of signs of loneliness and in whom I thought: yes, they just need the contact themselves”.^19^ Triangulation also disentangled that people use this type of volunteering to help them work through their own personal problems. Again derived from VFI *protective*, this applied to those who use this volunteer work to reintegrate into society after illness, burnout or dismissal. Lastly, people use this type of volunteering to explore different career options. That belongs to VFI *career*. This might happen when people get stuck in their careers and consider a change. One coordinator declared: “Some see it as, I want to make a switch and I am going to see what that is like, working with refugees or migrants”. They really see it as ‘I got stuck in my career and now I want something else’’.^20^

## Discussion

Focus groups with volunteers for refugees indicated that they value justice, and show a concern for unknown others. This study thus finds support for the social justice function, previously added as important motive to the VFI by Jiranek et al. (2013). It demonstrates their broader and more inclusive scope of justice (Kals & Strubel, 2017). Similar to Sandri (2018) volunteers were to a large extent motivated by feelings of compassion. This displays the altruistic, other-focused, humanitarian actions of the volunteers, as people “understand their ‘help’ as a humane duty to people in need” (Fleischmann & Steinhilper, 2017, p. 19). Note that we already argued in the theory section that *universalism* (and *benevolence*) is more or less represented in VFI *values*, however, given the results, the volunteers in our study do explicitly identify it as a distinct important value. Next, VFI *understanding* was very popular, thereby supporting the findings of Milan (2018) in her research among volunteers for refugees in Austria. An aspect that might explain the popularity of the *understanding* function is that volunteers for refugees are mostly higher educated (Kalogeraki, 2018). It may be possible that higher educated volunteers may appreciate more the learning effect in doing this volunteer work and also are more open to (learning from) other cultures because of their broader horizons (Wilson, 2012). Subsequently, this might partly explain the popularity of Schwartz’ *universalism* in this study, because of its reference to broadmindedness.

Results also revealed that volunteers mainly mobilize without career perspectives in mind. However, this outcome may be due to the composition of our sample. Most volunteers were retired, indicating that a resume was not important anymore. Triangulation, nevertheless, showed that some non-retired, older volunteers started volunteering to explore different career options. In this regard, VFI *career* can also be relevant for some older volunteers. The few younger people we had in our sample (like Monforte & Maestri, 2022a, 2022b) displayed the importance to volunteer in function of their CV. This supports the assumption derived from Musick and Wilson (2007) that young people are likely to volunteer out of career perspectives, especially when they are about to enter the labor market. It, however, raises the question why refugee support organizations have difficulties to attract and retain young volunteers. Della Porta (2018) suggested that heightened criminalization of refugees in which even practices of those offering support have come under attack (della Porta & Steinhilper, 2021b) may demotivate these young volunteers. Another clear result was the unpopularity of VFI *social*. Since we had an older sample, our findings contradict Dávila and Díaz-Morales (2009) who found that social motives were of importance among the oldest age volunteers. It should be noted, however, that some volunteers had experienced volunteering for refugees in the family sphere or have relatives who work in the field of immigration and got mobilized through these ways. These types of mobilization contain a social component. Additionally, some volunteers revealed that they were reluctant to talk about their volunteer work because they had unpleasant experiences. In these cases, (extreme) right-wing ideas of the other were the reason. These stories provide evidence that practices of humanitarian volunteers are increasingly under attack (della Porta & Steinhilper, 2021b) and might be understood in terms of shrinking civic space in light of ‘contentious solidarity’ (della Porta & Steinhilper, 2021a).

We also discuss the absence of political motivations, which are mentioned in more recent studies to be relevant for volunteering for refugees. In our study it was only mentioned once. Earlier studies show that political motivations become more important the longer people volunteer for refugees (della Porta & Steinhilper, 2021b; Hinger, 2016; Monforte & Maestri, 2022a; Sandri, 2018). This is related to (1) situations where volunteers face adversity and hostility toward their engagement and, consequently, politicize their motivations (Monforte & Maestri, 2022a); (2) interactions in the ‘spaces of encounters’ between volunteers (among themselves) and refugees (Fleischmann & Steinhilper, 2017) and (3) the highly politicized societal context with the subject of migration as dominant issue of political conflict (e.g., della Porta & Steinhilper, 2021b). Our sample, however, consisted mostly out of recently started volunteers who were activated by the opening of the temporary refugee camp. It is, therefore, quite possible that political motivations were not yet relevant to them. On the other hand, we identify the response to major events as portrayed in the media as a missing motive, which can easily be linked to the political motivation. But overall, this study found that political motivations were less important for the volunteers who recently started, which is in line with the literature (Eliasoph, 1998; Fleischmann & Steinhilper, 2017; Malkki, 2015; Monforte & Maestri, 2022a; Sandri, 2018).

Subsequently, triangulation demonstrated the importance of VFI *protective*. People volunteer for refugees to overcome their personal problems and to feel less lonely. Sandri (2018) demonstrated that volunteering for refugees indeed creates a significant space for sociality and new forms of community. Consequently, it offers “some degree of protection against loneliness, social isolation and asocial time” (Malkki, 2015, p. 134).

Next, four missing motivations were discovered. First, *exposure to controversial media* messages turned out to be an important motive. We define *media exposure* as a process where volunteer involvement is fueled by what people see and hear in the media. For example, people began volunteering for refugees after seeing the poignant images of dead toddler Alan Kurdi on a beach in Turkey in 2015 (Alcalde & Portos, 2018), or when former British Prime Minister David Cameron referred to refugees in Calais as a ‘swarm’ (Sandri, 2018). *Seeking for a meaningful role* in volunteering is a second missing motive. Not the least because many volunteers in the sample were retired and therefore were seeking for a meaningful daily activity. This closely relates to the role-substitution-perspective (Lancee & Radl, 2014), because retirees lose an important role in their lives and might try to retrieve another by volunteering for refugees. However, doing something meaningful also relates to VFI *values* (doing something for a cause that is important to me). So, it remains to be seen to what extent this missing motivation actually forms a different dimension as compared to the content of the VFI. Another missing motivation was the *pragmatism* of volunteers. Following Malkki (2015) and Monforte and Maestri (2022a), we refer to pragmatism as a perspective where volunteers focus on the tangible, visible and often quantifiable outcomes of their actions. Through this, they obtain their personal gratification (Krause, 2014). This immediate efficacy of their direct, hands-on aid, “can be identified as one of the main reasons why volunteers took part in [this] aid operation in such high numbers” (Sandri, 2018, p.74). It should be noted that pragmatism to some extent may be related to VFI *understanding*, as the tangible practice of skills is also central here. Considering *the emotional dimension*, the fourth missing motivation, volunteers mobilize because they identify emotionally with refugees based on (similar traumatic) biographical experiences in the past (Milan, 2018; Milan & Pirro, 2018). Volunteers are, for instance, emotionally involved because of a shared flight history (of a close relative) (Milan & Pirro, 2018). We should note that this also seems to contain a social component and therefore may be related to VFI *social* in some way. However, we suggest that these missing motivations should be tested in large scale surveys to test their potential as a motive for volunteering.

## Conclusion

This article examined the motives for volunteering for refugees and the extent to which the VFI is able to capture those motives. After all, a critique is that VFI functions are not exhaustive or fully comprehensive (Wilson, 2012). We find support for the added value of a social justice function, as previously added to the VFI by Jiranek et al. (2013). Next, four motivations that were missing from the theoretical framework we derived from earlier studies, arose from our data analyses. First, volunteers do this work because they search for meaningful roles. Second, volunteers have pragmatic reasons and engage in this work because of the tangibility and visibility of their volunteer actions. Third, exposure to controversial media messages fuels volunteer engagement. Emotions, being the final missing motivation, mobilize volunteers as they identify emotionally with refugees based on (similar traumatic) biographical experiences in the past. At first glance, the identified missing motivations seem distinct from the VFI. However, taking a closer look, aspects of some of the identified motivations seem to relate to the VFI. Therefore, we stress the need to include and test them as a new motivational framework for volunteering for refugees in large scale surveys in order to assess their prevalence and distinctiveness. This study supports the VFI’s emphasis on the motive that is summarized as ‘values’. People distinguish between different values that are all captured by the single dimension of VFI *values*. However, if the distinguished values would be operationalized by more items than is done in the VFI-instrument, we might arrive at distinctions in which Schwartz's *benevolence* and *universalism* also can be disentangled. For example, if in future research we have five separate items for each of the dimensions benevolence, universalism, compassion and helping others in need and we include those twenty items in a questionnaire and submit it to volunteers, do we still find one dimension of values or do we find those subdimensions? We therefore propose that future research should focus on this methodological matter to further improve the VFI *values* function. We also have to acknowledge, however, that the reduction in this study of the VFI scales to one item each might have affected the reliability of the results, since respondents were drawn, e.g., to one specific value on the VFI-*value* dimension. Next, we acknowledge that volunteers in our sample volunteered in a temporarily refugee camp that may reflect a response to act on a sudden crisis more explicitly than would have been found among volunteers who all volunteered for a longer period of time. We suggest to pose the question for future research whether motivations to volunteer are conditional on the context of the refugee-reception situation and the duration of volunteering. Further, there may have been sample bias since the data collection was in Dutch. Consequently, volunteers with a different migration background may have been excluded. Likewise, (former) refugees who volunteer themselves might have been excluded. We were thus not able to extend the research to refugees who volunteer themselves and possible inequalities in this process (Baillie Smith et al., 2022; Carlsen et al., 2022). Potentially, the absence of (former) refugees in the sample might have led to an underrepresentation of the emotional dimension as missing motivation to volunteer. Moreover, this study may have been subject to social desirability bias. The VFI is often used in anonymous questionnaires. It is possible that focus groups are more prone to social desirability bias. This study shed light on motivations to volunteer for refugees. We deem it important to get better understanding of the dynamics of volunteering as well and suggest to study whether there are factors during the process of volunteering for refugees that undermine or strengthen these motivations and how that matters for (dis)continuity in volunteering for refugees.

## Appendix

*A* Topic list volunteers and coordinators.

*Question 1* What are your motivations to do voluntary work for refugees?

There Exist Also Motivations Found in Earlier Research. Can You Indicate Whether These Apply to You?

Questionnaire motivations derived from theory.

Please indicate to what extent the motivations below play a role with regard to volunteering for refugees? Please circle your choice.

I do this voluntary work, because…

**Table:**

	Not important at all	Not important	Not important/not important	Important	Very important	I don’t know
It looks good on my resume	1	2	3	4	5	6
It relieves me of some guilt over being more fortunate than others	1	2	3	4	5	6
It let me learn things through direct, hands on experience	1	2	3	4	5	6
It makes me feel needed	1	2	3	4	5	6
People close to me do this type of voluntary work as well	1	2	3	4	5	6
I feel compassion toward people in need	1	2	3	4	5	6
I think it is important to respond to the needs of others	1	2	3	4	5	6
I want to ensure a better world for future generations people I don’t know	1	2	3	4	5	6
I want everyone to be treated justly, even	1	2	3	4	5	6
Refugees suffer emotionally	1	2	3	4	5	6
I think that refugees should be treated the same as others	1	2	3	4	5	6
I hope that my kindness now will be answered by some unspecified person in the case of future need	1	2	3	4	5	6
I want to care for my community	1	2	3	4	5	6

1. Unspecified person in the case of future need
2. I want to care for my community. 1 2 3 4 5 6

*Question 2* When looking at the questionnaire, which motivations are important for you?

*Question 3* Which motivations are not important?

*Question 4* Are there also motivations playing a role that were not on the list?

## Footnotes

1 Note that we do not intend to position the refugees as ‘passive aid receivers’ only, without acknowledging their own agency. Volunteer tasks were aimed at meeting the needs identified by refugees such as, providing legal and welfare assistance on the rights refugees have. Furthermore, daily interactions in the camp further give way to more equal (power) relations between volunteers and refugees (e.g. Monforte & Maestri, 2022b; Sandri, 2018).

2 It should thus be noted that even motivations that prove to be of little relevance for many people, might be very important for certain individuals.

3 Other distinguished values do not, or barely, apply to volunteering for refugees, as *achievement* and *power* values emphasize own relative success and control over others (Verkasalo et al., 2009). C*onformity*, *tradition* and *security* refer to values emphasizing self-restriction, preservation of traditional practices and protection of stability, whereas *hedonism*, *stimulation* and *self-direction* are ‘openness to change’ values which emphasize own independent thought and action (Verkasalo et al., 2009). Moreover, *benevolence* and *universalism* are most often linked with volunteering (Clary et al., 1998a). See Schwartz (1992) and Schwartz et al. (2001).

4 See for instance the similarities between VFI *values* item: *I feel it is important to help others* and *benevolence* item: *I volunteer, because it is important to respond to the needs of others*.

5 We argue that the other moral foundations do not, or barely, apply to volunteering for refugees, as *ingroup/loyalty* refers to dedication towards the own ingroup while refugees represent an outgroup (for at least most of the volunteers). This studies’ sampling strategy namely complicated the participation of (former) refugees (see limitations in the conclusion). For studies about refugee volunteering see Baillie Smith et al., 2022 and Carlsen et al., 2022 (these are also studies where ingroup/loyalty could apply to volunteering for refugees, in contrast to this study). Furthermore, *authority/respect* and *purity/sanctity* refer to moral standards that seem to lay outside the scope of volunteering for refugees. See Haidt (2013) for more background.

6 We acknowledge that a norm of *social justice* might be relevant as well (e.g. Musick & Wilson, 2007). The justice related motivation is, however, already included in Schwartz’ *universalism* and the *fairness/reciprocity* foundation of MFT.

7 Note that we obtained permission for this study after extensive screening by the University’s Social Science Ethics Committee. In addition, we sought permission from the organizations involved and formalized it. Next, all focus group participants and also the interviewees were briefed in writing in advance (active informed consent) about the background and purpose of the study. The Ethics Committee’s approval for this study is attached as Appendix D of the supplementary material (stored on Figshare.com).

8 Participant in focus group 1, Nijmegen, 23/11/2021.

9 Participant in focus group 7, online, 25/01/2022.

10 Participant in focus group 1, Nijmegen, 23/11/2021.

11 Ibid.

12 Participant in focus group 5, online, 22/01/2022.

13 Participant in focus group 1, Nijmegen, 23/11/2021.

14 Participant in focus group 1, Nijmegen, 23/11/2021.

15 Author interview with anonymous coordinator from Yalla Foundation, Nijmegen, 18/11/2021.

16 Participant in focus group 2, Nijmegen, 29/11/2021.

17 Ibid.

18 Participant in focus group 6, Nijmegen, 24/01/2022.

19 Author interview with anonymous coordinator from Vluchtelingenwerk, Nijmegen, 31/01/2022.

20 Author interview with anonymous coordinator from Vluchtelingenwerk, Nijmegen, 08/12/2021.
